# Plague in Arab Maghreb, 1940–2015: A Review

**DOI:** 10.3389/fpubh.2016.00112

**Published:** 2016-06-03

**Authors:** Maliya Alia Malek, Idir Bitam, Michel Drancourt

**Affiliations:** ^1^Aix Marseille Université, URMITE, UMR 63, CNRS 7278, IRD 198, INSERM 1095, Faculté de Médecine, Marseille, France; ^2^Laboratoire Biodiversité et Environnement: Interactions Génomes, Faculté des Sciences Biologiques Université des Sciences et de la Technologie Houari Boumediene, Bab Ezzouar, Algeria

**Keywords:** *Yersinia pestis*, plague, outbreak, foci, Maghreb Arab

## Abstract

We reviewed the epidemiology of 49 plague outbreaks that resulted in about 7,612 cases in 30 localities in the Arabic Maghreb (Mauritania, Morocco, Algeria, Tunisia, Libya, and Egypt) over 75 years. Between 1940 and 1950, most cases recorded in Morocco (75%) and Egypt (20%), resulted from plague imported to Mediterranean harbors and transmitted by rat ectoparasites. By contrast, the re-emergence of plague in the southern part of Western Sahara in 1953 and in northeast Libya in 1976 was traced to direct contact between nomadic populations and infected goats and camels in natural foci, including the consumption of contaminated meat, illustrating this neglected oral route of contamination. Further familial outbreaks were traced to human ectoparasite transmission. Efforts to identify the factors contributing to natural foci may guide where to focus the surveillance of sentinel animals in order to eradicate human plague, if not *Yersinia pestis* from the Arab Maghreb.

## Introduction

Plague, a deadly zoonosis caused by the Gram-negative bacterium *Yersinia pestis*, historically affected the Arab Maghreb (comprising Mauritania with the Western Sahara, Morocco, Algeria, Tunisia, Libya, and Egypt) over a period of at least two millennia (1). This is a review of plague cases from 1940 until the present day in the Arab Maghreb, with a view to restricting the evolution of the disease in this region and to identify features differentiating its epidemiology in the Arab Maghreb from other regions of the world (2).

## Methods

Information provided in this review has been compiled from the World Health Organization’s weekly report, information issued by the Algerian Ministry of Health, and published papers retrieved from the NCBI’s PubMed. We also reviewed the Bulletin de la Société de Pathologie Exotique published in French and communications by professors M. Baltazard and H. H. Mollaret from the Pasteur Institutes. We also consulted gray literature to supplement officially reported data regarding the distribution of plague outbreaks, foci, and the movements of the affected populations. Those data were collected from testimonies from local populations. The fact that plague records were not as systematic as they were over the same period in the US, necessarily limit available data (2).

## Results

### Descriptive Epidemiology

Between 1940 and 2015, a total of 49 plague outbreaks resulted in 7,612 cases in 30 localities in the Arab Maghreb (Video S1 in Supplementary Material). Reports made in 1940–1945 during the Second World War (WWII) did not detail the age, sex, and mode of contamination of patients. After 1945, plague cases were more accurately reported. We distinguished three periods between 1940 and 2015 with identifiable epidemiological patterns.

### First Period, 1940–1950

During WWII, plague affected all the Arab Maghreb countries except for Mauritania and accounted for 6,801/7,612 (89%) cases. Morocco alone recorded approximately 5,500 (71%) cases in the regions of Chaouia (20%), Agadir (14%), Marrakech (12%), Rabat, Doukkala, and Port Lyautey (1%) (3). In April 1940, preceded by a murine outbreak, plague affected Agadir and spread to Marrakech where it caused 498 cases 5 months later. Two outbreaks in 1941 in inland south Morocco suggested that they resulted from endemic foci (4). In 1942, 25 cases notified in Casablanca probably resulted from the train transport of grains by rail from the southern foci where 583 cases were declared. In 1943 and 1944, plague spread to Rabat and caused 393 cases in Port Lyautey and 227 cases in Marrakech, respectively. Early in 1944, Casablanca suffered another episode of 79 bacteriologically confirmed cases; followed by an 828-case devastating episode that was the last to be recorded in Morocco. In Algeria, 8 cases were recorded in Algiers in the early 1940s, 94 cases in 1944, and 11 cases in 1945. The same year in Oran, where the French Nobel-prize winning author Albert Camus located his novel “La Peste” (The Plague), six pneumonic plague cases occurred (5). Another two cases in 1946 and six cases in 1950 (3) seemed to have been imported cases except for two local cases (6). In Tunisia, 12 cases were recorded in 1940 and 1 in 1941. In August 1944, a fatal index case was confirmed by blood smear examination and isolation of *Y. pestis* in laboratory animal at Ferryville (currently known as Menzel Bourguiba). A final 37-case outbreak included 25 European patients. In Libya, 12 confirmed and 2 suspected cases were noted in 1939–1943 in a locality 12 km from Tripoli (7). In Egypt, 452 cases were recorded in 1940 in the province of Assiut in Upper Egypt and some sporadic cases were also reported in Port Said. In November 1943, an outbreak was observed in the Suez Canal area; and in the Ismailia district and Port Said in 1944 accounting for a total of 862 cases. Plague reappeared in February 1945 causing 19 cases in ports along the canal to gradually dwindling with two cases in 1946. In January 1947, a 15-case outbreak took place in Alexandria after a 12 years gap and was the last reported outbreak in Egypt. It was reported that during this period the plague had essentially port profile in contrast to previous outbreaks that took place in rural areas (8).

### Second Period, 1951–2015

In Mauritania, plague increased to a cumulate effective of 3%. In January 1953, a plague outbreak may have taken place in the Río de Oro, south-western Sahara but this episode was not microbiologically documented and was not declared to the WHO (9). According to the Mauritanian Ministry of Health, 74 human cases were identified between Boulanouar and Bir Quendouz after similar human cases had been observed in 1951 in Bir Moghrein, a quasi desert region in north Mauritania (9). Ten years later, four cases were diagnosed at Bir Igueni, an area inhabited by 3,000 nomads (9). Investigation uncovered nine deaths in the previous 4 months. Nine fatal cases in the Nasri area also remained unreported to the WHO. Cases, without bacteriological confirmation and medical supervision, were collected by interviewing nomads who reported the first fatal case of plague in a woman from the Rio de Oro. Other cases include another woman, an 8-year-old girl and the grave digger, who had been in close contacts with the index case. On 16 October, at Port Etienne, five cases were reported of family members with a cervical tumefaction. Of them, four died. The doctor P. Bres reported a total of 11 deaths for this episode (9). Four cases were declared at the Al Mounek camp and two others in mobile camps were identified one further north, at Bir Tenchi and Aguedat Iguenine, along with sick cattle. Investigation by M. Baltazard and J. M. Alonso discovered that plague had been moving from southern Morocco to Mauritania for years (9). A 23-case outbreak reported the same year in northwest Mauritania was microbiologically documented (9). At the same period at Fort Coppolani, an outbreak included a dozen cases and one deceased woman presenting buboes without bacteriological identification.

In Libya, after a 30-year gap, an 18-case outbreak occurred in the Nofila area in l972. In 1976, an outbreak occurred in the northeast at the village of Al-Azzizat. Near Tobruk in the village of Krom-el-Kheil, five family cases occurred. All recovered after early antibiotic treatment but only four were plague positive. In Al-Azzizat, one human and four goats cases were reported. In January 1977, an 11-case outbreak took place in Jadu in southwest of Tripolitania (10). In September 1984, eight bubonic plague cases occurred at two locations 25 and 60 km from Tobruk at the Egyptian border. Libya declared an episode in June 2009 in Betnane, a semi-nomadic area in the coastal town of Tobruk (11). Another possible outbreak of plague comprising more than 20 cases occurred at Tobruk during the Libyan revolution in May 2011, but due to the political situation of the country no scientific evidence was provided.

No natural plague focus had ever been described and confirmed in Algeria until the outbreak in Kehailia and neighboring villages, 30 km southwest of Oran on 22 June 2003 (12). Indeed, after a 53-year gap, on 4 June the University Hospital Center of Oran received an 11-year-old boy with signs of septicemia. A dozen adults with similar symptoms were diagnosed with bubonic plague in the area of Oran with nine urban and rural cases, one case in Mascara and two cases in Ain Temouchent (13). In July 2008, a new episode took place for the first time in the Laghouat area in a nomad camp in Thait El Maa with four cases (14). This was the last episode of plague in Algeria until the present day.

### Clinical Features and Outcomes

While penicillin G was shown to be ineffective, sulfonamides and serotherapy allowed for effective prophylaxis (15). Streptomycin was first used in 1947 during the Haifa outbreak (16). Accordingly, we estimated mortality during the first period (1940–1950) to be of 63% significantly higher than the 28% mortality during the 1951–2015 periods (*P* < 0.05). Clinical descriptions indicated that 87% patients had inguinal or cervical buboes, 7% had a primary pulmonary plague, and 6% had a septicemic form of plague. Mauritanian cases described in 1953, 1963, and 1967 were all sporadic cases that occurred suddenly in a limited region and were described as cervical bubonic plague (9). Inhabitants observed an increase in the commensally rodent population, but no unusual rodent mortality before the outbreak. Furthermore, the appearance of cases in a location which is 100-km distant does not mean expression of different foci at the same time but the same focus because the isolates all belonged to the same strain and are classified in clusters that are different than those of America and Africa suggesting a non-imported plague (13). In that region, close contacts with *Meriones* could be explained by lack of food that leads the rodent to get into seeds’ reserve. In the last Oranian outbreak, all 12 confirmed cases presented buboes. Plague was confirmed by a rapid diagnostic test (17) and *Y. pestis* was isolated from bubo or blood in six patients. In Laghouat, four people had swollen lymph nodes, one patient who developed pulmonary form died (14). The other three patients were treated with doxycycline, rifampicin, and intramuscular gentamicin after bacteriological confirmation of plague by culturing *Y. pestis* from bubo aspirate. Rats or *Y. pestis*-susceptible rodents were absent in the nomads’ environment. In Libya, in 2009, the first case was a child with pneumonic plague, who died; two family members of the child and two women presented a bubonic form (11). In the Maghreb episodes, a few *Y. pestis* isolates have been fully characterized but it was confirmed that strains responsible for the 2003 and 2008 episodes in Algeria were of the Orientalis biotype as were four strains isolated in 1940–1945 in Morocco and Algeria (14, 18, 19); whereas strain responsible for the 2009 episode in Libya was of the Medievalis biotype (19). Further pulsotype analyses indicated that the 2009 Libya episode was most likely due to a unique strain contrary to the 2003 Algeria episode that was most likely due to several, closely related strains (19).

### Sources of Human Plague

Both wild rodents and domestic mammals have been sources of zoonotic plague in the Arab Maghreb. In Mauritania, all the cases were accompanied by sick cattle and significant rodent mortality. *Psammomys obesus, Gerbillus gerbillus*, *Gerbillus pyramidum*, and *Gerbillus nanus* are major reservoirs (20). *Jaculus jaculus*, carriers of *Synosternus cleopatrae*, are of particular interest due to the density that could be involved in the epidemic process; and the fact that their movements exceed that of other gerbils (21). In Morocco, *Rattus norvegicus* is found in the north, and *Rattus rattus* and *Rattus rattus alexandrinus* in the south. Within the country, many *Meriones* jirds were also found. The most common flea was *Xenopsylla cheopis*. In Algeria, as in Mauritania, plague-resistant *Meriones shawi* carrying the *Xenopsylla ramesis* flea contribute to plague persistence. As for the 2004 outbreak, it was observed that the villagers had carried out a pest control campaign around the time of the fatal index pediatric case. Moreover, villagers suffered from poor housing with *R. norvegicus* infestation in wheat storage areas. From September 2004 to May 2005, our team found a 21% prevalence of *Y. pestis* in 90 *X. cheopis* fleas collected from *R. rattus*, *R. norvegicus*, *Mus musculus*, and *M. spretus* in this area (12). Moreover, *Y. pestis* was cultured from two *M. shawi* animals, a plague-resistant rodent species captured in Laghouat in January 2009, suggesting a new plague focus in Algeria (14). This observation was confirmed by a further field investigation from 2009 to 2012, capturing 237 rodents revealing the persistence of plague in Oran and Laghouat and finding three new plague foci at Cap Djinet, Biskra and M’Sila (22). In particular, the *Apodemus sylvaticus* wood mouse positive for *Y. pestis* for the first time in addition to *R. rattus*, *M. shawi, P. obesus*, *Mus spretus*, and *Crocidura russula* (22). In Tunis, there was a predominance of *R. norvegicus* where 75 were infected, along with *R. rattus alexandrinus* black rats (29 infected), *R. rattus* (13 infected), *Mus gentilis*, and *Mus azoricus* mice of which, respectively, 9 and 3 were found to be pestiferous (3). The predominant flea was *X. cheopis*, while *Leptopsylla segnis* and *Nosopsyllus fasciatus* appeared rarely. In Libya, *G. gerbillus* and *M. shawi* were the most common rodents and *G. gerbillus* were captured inside nomad tents (3). *Meriones libycus* is a more widespread species and is comparatively resistant to plague. It has also been found to be seropositive for plague. In the north plague focus, *M. libycus, Meriones caudatus, Psammomys obesus*, *and M. shawi* were present and carrying *X. ramesis*, *X. cheopis*, *Xenopsylla taractes*, and *Nosopsyllus henleyi* fleas. In Egypt, *R. norvegicus* in ports, *R. rattus* at warehouses, and *Acomys cahirinus* present at along the Suez Canal were in close contacts with populations (3). *X. cheopis* is the most common flea followed by *L. segnis*. Infestation of *R. rattus* by fleas was one of the principal factors for the high prevalence of plague in Upper Egypt where it was particularly to be found.

In these situations, controlling wild rodents and their flea ectoparasites proved to be more difficult than controlling the commensally species due to difficulties in locating burrows and runways and the wide dispersion of rodent populations rendering difficult to precisely decide on the limits of the area to be treated. Before the appearance of DDT and in some areas to over 80 years, flea and rodent control was carried out by fumigating burrows with cyanide gas through insufflations of HCN dusts or granules. While the results of fumigation are often dramatic, this method had several shortcomings. In large burrow systems, the fumigant was often too light to reach all parts of the burrow system and rodents could escape its effects. Therefore, 10% DDT dust was one of the most common and effective compounds used in rodent flea control programs. However, widespread emergence of insecticide resistance in populations of several important vectors, including *X. cheopis*, and the increased concern over environmental contamination, alternative compounds have been used in the more recent episodes. Most of these compounds are effective against both adult and larval fleas. These alternative insecticides include the organo-phosphorus, carbamate, pyrethroid, and insect growth regulator compounds shown to be effective in field trials. The powders applied on the slopes or in the holes (commensally rodents) or inside burrows (wild rodents) proved to be effective against flea vectors. Rodents through spots powder on their slopes or out of their burrows pick insecticide powder in their fur and spread when they lick themselves, thus killing ectoparasites fleas. In modern-day Maghreb, WHO-recommended insecticides include the pyrethroids with residual effect, such as Deltamethrin powder and liquid formulations of permethrin.

Semi-domestic and domestic animals are another source of plague. Buboes are seen in domestic mammals, such as cats, dogs, and camels (23). In camels, the principal companion of nomad tribes, buboes have long been known as “gudda” in the Arabian Peninsula and have been linked to human plague bubo by nomads. Camel plague has been described by Sotnikov in 1974 in Africa, Eurasia (USSR), Asia (Mongolia, China, India), and the Middle East (Iran, Iraq) (24). Camels may present the three forms of plague and die within 20 days after an incubation period of 1–6 days. Not only camels but also gazelles, goats, sheep, and hares die inexplicably and although not forming part of the cycle of plague were described as being responsible for contamination (3, 25). Four patients with adenitis were in close contacts with cattle, including five sheep with superficial adenitis in the Al Mounek camp (9). In 1976, in Libya four patients contracted plague 4 days after slaughtering and skinning a camel. The camel was eaten by some villagers after having been in contact with it, including one who resold the meat. Seven adults exhibiting a serologically confirmed bubonic plague were also reported and the reseller’s daughter presented with groin bubo. Villagers testify that the slaughter took place following an illness contracted by the camel that presented a swollen neck gland but no study states whether it was confirmed as plague positive or not. In Krom-el-Kheil, the outbreak started by a father who killed and skinned a sick goat 2 days before his admission to hospital. The goat skin was kept and treated by the woman at home, where recent and older dead rats were found. It was, therefore, supposed that rat flea were responsible for the goat infection. In Al-Azzizat, a 12-year-old boy, who had also skinned a sick goat, contracted the disease and recovered with anti-biotherapy. Four goats from the herd were tested and one of them was revealed as being seropositive to *Y. pestis*. In January 1977, an outbreak in Jadu in southwest of Tripolitania, involved 11 cases following the slaughter of a dying sheep, confirmed by *Y. pestis* isolation. In April–May 1967, an epizooty of rodents and gazelles and one child died of adenitis in Aguedat Iguenine was reported in a permanent nomad camp in Mauritania (9). Four months before the first outbreak, fleas in tents and the absence of dead rodents were observed with deaths in camels, cattle, sheep, goats, and even donkeys. Human cases suffering from bubonic plague may have resulted from contacts with sick camels that had been killed. Camel buboes cultured *Y. pestis*, Orientalis biotype but plague remained undeclared to the WHO (9). In 32 patients, plague could be traced to direct animal contacts with goats, sheep, and camels. In particular, eating camel meat recovered from a sick animal was documented in Mauritania and Libya for the second period. In addition, the epizooty in camels had resulted in human cases by affecting nomadic tribes. There is one confirmation of a published report of oral contamination. Christie et al. (10) proposed an oral transmission route of in the 1976 Libyan outbreak. Goat and sheep are also considered to be sentinel animals, indicating plague in a given focus (3). The 1977 outbreak in Libya confirmed the high risk of direct contact with carcasses from livestock infected with plague. In addition to their migrant lifestyle leading to close contacts with rodents and thereby promoting the spread of the epidemic to other regions, some nomads’ tribes consumed dried rodent meat and traded in rodent furs (26). The majority of plague foci in the Maghreb are located in sub-desert nomadic regions. To the east there is the Libyan focus and to the west there is the northeast focus of Mauritania. In low-lying areas, where the habitat is particularly favorable both to the survival of the plague bacillus by the presence of sensitive rodent species and the availability of watering points in demand by migrants for stopovers and the presence of plants that camels fed upon (21).

Infected patients were a last source for secondary cases in four outbreaks. In seven patients, primary pneumonic plague could not be traced to direct air-borne transmission from an index case resulting from this route of contamination. We observed a significant involvement of human ectoparasites, such as *Pulex irritans* fleas and *Pediculus humanus* lice (27, 28). Experimental research undertaken in Morocco showed the possible transmission of plague by human ectoparasites, fleas, and body lice stating that without human ectoparasites, bubonic plague epidemics are not possible (29). Moreover, careful investigation of cases means that family cases could be described and that transmission from person to person by human ectoparasites could be highlighted (30).

## Discussion

Despite limitations due to under-reporting of plague in Mauritania in 1963–1967, and in Libya and Egypt in 1984 to avoid isolation and quarantine, and poor clinical descriptions of cases during the first period, we are confident that data reported here on plague over 75 years in the Arab Maghreb are reasonably sound enough to draw a picture of an evolving situation and to identify prospects for the next decade. The epidemiology of plague dramatically changed over 75 years in the Arab Maghreb with a sharp decrease in its overall prevalence from 6,801/22,946,800 inhabitants in the 1940s to 43/92,586,424 inhabitants in the 2000s. Likewise, prognosis dramatically changed with mortality taking a sharp 0.02% decline to a residual mortality of 4.10^−5^ in the 2000s.

Moreover, plague epidemiology changed from mixed rural and urban epidemics to rural epidemics involving nomads who currently remain the sole populations to be affected by deadly plague. Nomads mainly acquired plague through close contacts with domestic goats and camels. In particular, the consumption of poorly cooked meat from slaughtered sick animals is a source of plague for these populations; illustrating that deadly plague could be transmitted orally, as also described in Saudi Arabia in the form of severe pharyngitis in 1994 (31) and in Jordan in 1997 (32). Although this route of contamination has been neglected, animal models show that *Y. pestis* could cause deadly septicemia after intragastric inoculation, without stool excretion (33). This situation is not surprising considering that *Y. pestis* was shown to have evolved from *Yersinia pseudotuberculosis*, a pathogen responsible for digestive tract infection, after chromosome reduction and acquisition of three plasmids (34). Accordingly, *Y. pestis* retained the *Yersinia* genus capability to enter the digestive tract, further acquiring the capability to cross the digestive tract barrier to provoke deadly septicemia. These observations should be taken into consideration for further risk assessments of human plague.

The fact that plague re-emerged in the very same location after decades of absence (11, 12, 14), and was genetically documented as being local one (19) along with continuous documentation of zoonotic plague illustrate the presence of plague foci in the Arab Maghreb. Evidence that *Y. pestis* persists in the soil under natural (35) and experimental (36) conditions suggests that plague foci are telluric, where burrowing mammals could be infected by contacts with infected soil. Accordingly, nomads used to avoid the regions where plague cases had occurred, described as “cursed areas.” Respect for this rule even led people to believe that plague had been eradicated. This situation in the Arab Maghreb is similar to that reported in the US (2) but contrasts sharply with the situation in neighboring Europe. Both Europe and the Arab Maghreb were exposed for two millennia to three historical plague pandemics, and recent genotyping data suggest that it may have established stable reservoirs in Europe during the fourteenth to seventeenth century epidemics (37). Nevertheless, plague did not established stable foci in Europe beyond historical period and no autochthonous case for 50 years (38). Understanding the factors contributing to such contrasting situations may contribute to the overall understanding of the disease and its prevention.

In conclusion, the unique epidemiological and clinical features of plague in the Arab Maghreb create a comprehensive view of plague as being a deadly infection residing in telluric foci that are sources for zoonotic plague transmitted to populations by direct and ectoparasite-borne contacts with infected animal and the consumption of infected meat. Primary digestive plague is a neglected yet deadly form of infection. In the Arab Maghreb countries, efforts should be made to understanding factors conditioning telluric plague foci and to strengthening surveillance of sentinel animals and ectoparasites. These efforts should be pursued to strengthen prevention in nomadic populations, including hygiene in homes and cooking.

## Author Contributions

MM reviewed data and drafted the manuscript. IB drafted the manuscript. MD decided of the topic, reviewed data, and drafted the manuscript.

## Conflict of Interest Statement

The authors declare that the research was conducted in the absence of any commercial or financial relationships that could be construed as a potential conflict of interest.
